# A Cost-Effectiveness Analysis of Blended Versus Face-to-Face Delivery of Evidence-Based Medicine to Medical Students

**DOI:** 10.2196/jmir.4346

**Published:** 2015-07-21

**Authors:** Stephen Maloney, Peter Nicklen, George Rivers, Jonathan Foo, Ying Ying Ooi, Scott Reeves, Kieran Walsh, Dragan Ilic

**Affiliations:** ^1^ Monash University Frankston Australia; ^2^ Monash University Caulfield Australia; ^3^ Centre for Health & Social Care Research, Kingston University & St George’s, University of London London United Kingdom; ^4^ BMJ Learning London United Kingdom; ^5^ Monash University Melbourne Australia

**Keywords:** evidence-based medicine, economic evaluation, eLearning, medical education

## Abstract

**Background:**

Blended learning describes a combination of teaching methods, often utilizing digital technologies. Research suggests that learner outcomes can be improved through some blended learning formats. However, the cost-effectiveness of delivering blended learning is unclear.

**Objective:**

This study aimed to determine the cost-effectiveness of a face-to-face learning and blended learning approach for evidence-based medicine training within a medical program.

**Methods:**

The economic evaluation was conducted as part of a randomized controlled trial (RCT) comparing the evidence-based medicine (EBM) competency of medical students who participated in two different modes of education delivery. In the traditional face-to-face method, students received ten 2-hour classes. In the blended learning approach, students received the same total face-to-face hours but with different activities and additional online and mobile learning. Online activities utilized YouTube and a library guide indexing electronic databases, guides, and books. Mobile learning involved self-directed interactions with patients in their regular clinical placements. The attribution and differentiation of costs between the interventions within the RCT was measured in conjunction with measured outcomes of effectiveness. An incremental cost-effectiveness ratio was calculated comparing the ongoing operation costs of each method with the level of EBM proficiency achieved. Present value analysis was used to calculate the break-even point considering the transition cost and the difference in ongoing operation cost.

**Results:**

The incremental cost-effectiveness ratio indicated that it costs 24% less to educate a student to the same level of EBM competency via the blended learning approach used in the study, when excluding transition costs. The sunk cost of approximately AUD $40,000 to transition to the blended model exceeds any savings from using the approach within the first year of its implementation; however, a break-even point is achieved within its third iteration and relative savings in the subsequent years. The sensitivity analysis indicates that approaches with higher transition costs, or staffing requirements over that of a traditional method, are likely to result in negative value propositions.

**Conclusions:**

Under the study conditions, a blended learning approach was more cost-effective to operate and resulted in improved value for the institution after the third year iteration, when compared to the traditional face-to-face model. The wider applicability of the findings are dependent on the type of blended learning utilized, staffing expertise, and educational context.

## Introduction

Evidence-based medicine (EBM) combines the best available evidence with clinical expertise and patient values [1] and is core to many medical programs worldwide [2-5]. There is an imperative to increase the number of competent EBM trained practitioners. Medical practitioners who are competent and confident in applying EBM possess a powerful tool to inform their decision making. EBM competencies also provide the ability to facilitate life-long learning, as clinicians are able to ask effective clinical questions, acquire information through emerging research, appraise its quality and relevance, apply evidence to practice, and assess its impact [1].

Teaching and learning is undergoing a cultural change with the expansion of student-centered learning, including the push for flipped teaching, peer-assisted learning, increased use of virtual learning environments, and the use of simulation. Research has shown that Web-based learning results in improved outcomes when applied to health professional education [6], with studies focusing on clinical disciplines within medicine reporting an increase in student self-efficacy, knowledge, and self-directed learning [4,7-9]. However, due to the variety of online learning applications and education provider contexts, there is an added need for detailed and robust studies [10], as uninformed transition of learning material to an online environment can have a negative impact on educational outcomes [11]. Online education, or its many variations (eg, eLearning, Web 2.0), appears to hold great promise for addressing the accessibility and efficiency of education, yet currently there is a lack of evidence to inform educators and learners as to the most effective methods of teaching EBM to medical students.

An investigation into the effectiveness of implementing a blended learning (BL) versus a traditional face-to-face (F2F) learning approach of teaching EBM to medical students was conducted by Ilic et al [12]. This multicenter international study used validated outcome measures of EBM competency to determine that BL is no more effective than F2F at increasing medical students’ knowledge and skills in EBM. These authors concluded that the BL approach was significantly more effective at increasing student attitudes toward EBM and self-reported use of EBM in clinical practice. Although their study looked at competency, attitudes, skills, and behavior, the missing piece of the puzzle, as acknowledged by the authors, was a measure of cost-effectiveness to help facilitate sustainable adoption of the approach.

The cost and value of teaching and learning practices in medical education have a direct impact on the accessibility of education, the efficiency and quality of education, and the productivity of our health workforce [13-15]. Cost-effectiveness analysis allows decision makers to decrease the risks of implementation from both a financial perspective and the perspective of maintaining quality of education, thereby facilitating adoption [16]. Considerations of cost and value are most commonly known for their application to pharmaceutical interventions: impact of drug A versus drug B, with consideration of the drug’s effectiveness, side effects, and value for money. Pharmacists can still stock the most expensive choices, and consumers can still purchase them; however, these are informed decisions based on the evidence. The same principles should apply to education. For an educator to effectively review their practices and pedagogy, they must consider the learning experience and learning outcomes alongside measures of cost and value [17].

Current literature on the cost and value of technological innovations in education is divided, with some studies supporting its cost-effectiveness [18], and others indicating that costs are higher as a result of increased resource development time and need for technological support [19,20]. Economic analysis for health education has primarily focused on telemedicine technology, medical reviews by remote physicians [21], or has been concerned with the cost-effectiveness of modalities for patient education [22]. Previous cost studies on BL approaches in health professional education have typically been conducted on short courses with small sample sizes [18,19] or were unable to determine the cost-effectiveness relationship [23,24]. Thus, there is considerable doubt as to whether existing literature could be generalized to the core-teaching content of a contemporary medical program.

This paper presents the findings from a study that aimed to compare the cost-effectiveness of a face-to-face approach and blended learning approach for EBM training within a medical program—training that has rigorously evaluated effectiveness as tested within a randomized controlled trial (RCT).

## Methods

### Design

The economic evaluation was conducted as part of an RCT comparing the EBM competency of medical students who participated in two different modes of education delivery [12]. The attribution and differentiation of costs between the interventions within the RCT, in conjunction with measured outcomes of effectiveness, enabled a cost-effectiveness analysis to be applied.

### Trial Participants, Methods, and Results

The fundamental elements of the RCT have been reported to provide context for the application of the cost-effectiveness analysis. Further detail on the methods and results of the RCT are available within a published paper [12] and pilot study [4].

A multi-campus study was performed with medical students enrolled in the Bachelor of Medicine, Bachelor of Surgery course at Monash University, Melbourne and Malaysia, including both undergraduate and post-graduate students. Participants were third-year medical students, who were all entering their first year of clinically based training and first year of formal EBM training. The EBM unit is integrated within the medical curriculum and is equivalent to a 6-credit point unit. A total of 497 students were randomized to receive EBM teaching via either the incumbent face-to-face approach (F2F) or the blended learning approach (BL). Students randomized to the intervention group received the same theoretical concepts taught in the control group, but in a BL approach. The BL approach to teaching EBM integrated (1) classroom activities (lecture/tutorial) with (2) online and (3) mobile learning as described in Table 1.

**Table 1:** Detailed description of the DL and BL teaching approach to EBM with associated costs.

Teaching	Description	Contribution to costs
F2F classroom activities	Student receive 10 x 2 hour classes. Each class starts with the tutor presenting on the EBM content for the session. Students then complete small group tasks and participate in large group discussions lead by the tutor.	F2F classroom activities contribute to staff preparation and teaching time, as well as space charges.
BL classroom activities	At the start of semester, students receive a 4-hour workshop on EBM concepts and an introduction to the BL format. The remaining classes are run in small group format with topics for discussion set by the tutor. Tutors facilitate peer-to-peer learning with a quasi-journal club delivery method where students are assigned topics to investigate during the week. Students then report on their findings in the next session.	BL classroom activities contribute to staffing preparation and teaching time, as well as space charges. Staff have a less active role in the activities, acting as facilitators than tutors. Thus, compared to DL classroom activities, the preparation and teaching costs are lower.
BL online activities	Online activities include a YouTube channel and an online Monash University library guide. The YouTube channel has 11 online lectures with an average length of 17 minutes [25]. The library guide indexes online resources (eg, databases, textbooks, and guidelines) and instructs students on how to use them [26]. Students are sent online activities to complete prior to the classroom session and can also use the online resources to assist learning during the week.	The YouTube channel was developed by Monash University staff solely for the teaching of the EBM unit, making up a large portion of the transition costs. Library staff generate online guides for many subjects. The guide used for EBM teaching existed prior to the BL transition. Thus, costs were attributed to designing activities using the online guide resources, but not the creation of the online guide.
BL mobile learning	Mobile learning occurs on the wards, where students interact with patients during their existing day-to-day “beside teaching” schedule—a method previously piloted [4]. Based on their assigned topic for the week, students are required to identify a patient, take a detailed history, and apply the principles of EBM relevant to the patient.	Mobile learning is completely student self-directed. There were no costs associated with mobile learning factored into the cost model.

A total of 147 (29.6%) of the 497 students completed the follow-up assessments on EBM competency and attitudes. EBM competencies were assessed using the validated Berlin questionnaire [27]. Students’ self-efficacy, attitudes, and behavior were also assessed. EBM competency did not differ significantly between students receiving the BL approach versus those receiving the F2F approach: mean difference -0.68, 95% CI -1.71 to 0.34, *P*=.19. Although student ratings of self-efficacy, attitudes, and behaviors all displayed a significant preference for the BL model. In total, 74 students completed the F2F model of training, with a mean score of 7.98 (SD 3.35), and 73 students completed the training via the BL approach, with a mean score of 8.67 (SD 2.96).

### Economic Analysis Procedure

**Figure 1:**
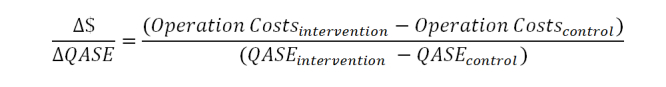
Equation for the calculation of the incremental cost for each quality-adjusted student educated (ICER).

The following analysis was applied from the perspective of Monash University, measuring the cost of training student clinicians against their self-reported level of EBM competence. The primary outcome was student competency in EBM, measured 1 month after the teaching activities, using the validated Berlin Questionnaire. We calculated the cost-effectiveness for each course delivery method by first determining the quality of students’ education with each method, known as quality-adjusted students educated (QASE), using the formula QASE = number of students educated x the group’s average rating on the Berlin Questionnaire. In this approach, the reported average rating was used as a surrogate for measuring the improved ability of the total cohort of 497 students for each teaching approach. QASE is the measurement of effect in the incremental cost-effectiveness analysis. Cost-effectiveness was calculated using the incremental cost-effectiveness ratio (ICER), which measures cost per QASE (Figure 1). The ICER is reflective of the ongoing operational costs and does not include the initial transition cost in its calculation. Thus, the results represent the cost-effectiveness of the second iteration and onwards only.

Two rounds of ICER calculations were completed. First, an ICER comparing F2F to BL was calculated, establishing the hierarchy of cost-effectiveness. There were four possible outcomes of this analysis: (1) BL is more costly and more effective than F2F, (2) BL is more costly and less effective than F2F, (3) BL is less costly and more effective than F2F, and (4) BL is less costly and less effective than F2F [28].

The World Health Organization recommends that cost-effectiveness comparisons should be carried out against a common baseline as this is more comparable across populations and studies [29]. Thus, in the second round of ICER calculations, F2F and BL were independently compared to no EBM training. As there are no pre-test Berlin scores from the RCT, baseline scores from third-year medical students with no prior EBM training (mean 4.2, SD 2.2) from the Berlin Questionnaire validation study by Fritsche et al were used [27]. Independent ICER calculation of F2F and BL compared to baseline calculates the cost per student per increase in QASE. Due to commercial sensitivities, these values were compared and the percentage difference reported.

**Figure 2:**
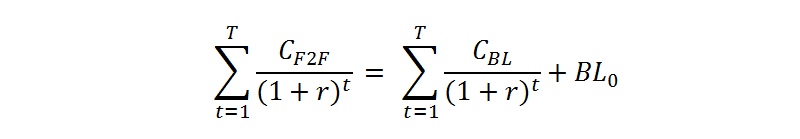
Equation for the PV break-even calculation where C=cost of teaching method, r=discount rate, t=number of years, and BL_0_=cost of transitioning to BL.

The sunk cost of transitioning to a BL format was not included within the ICER, as is typical within economic analyses. However, the transition costs are reported separately, due to its importance to decision makers considering implementation or adoption of similar pedagogy. A further present value (PV) break-even analysis incorporating transition costs was calculated using a real discount rate of 4% (Figure 2). Break-even analysis calculates the point in time at which the total running cost of the F2F approach equals the total running cost of the BL approach plus the cost of transitioning to BL. PV accounts for the time preference of money, allowing for present day comparisons to be made on future cash flows. Subsequent iterations of the program are assumed to occur at 1-year intervals. Due to commercial sensitivities, PV values are expressed as the difference between teaching methods.

### Measurement of Cost

**Figure 3:**
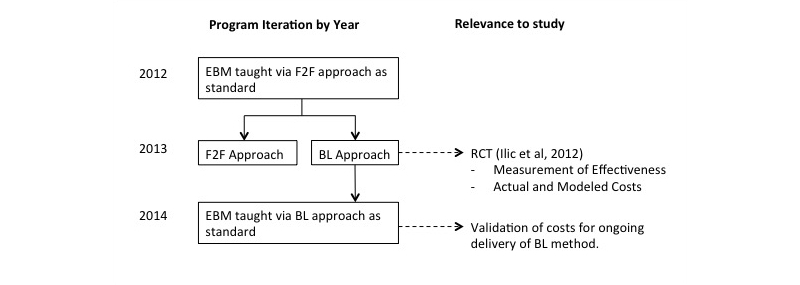
Program iterations and outputs relevant to past and current research.

The BL arm of the RCT has continued as the preferred method of delivery of EBM with the medical program. Costs were modeled on the 2013 RCT, and the assumptions relating to the ongoing costs were validated within the 2014 iteration of the program. The course iterations and outputs as they relate to the analysis are shown in Figure 3.

Cost categories considered within the analysis included (1) transition costs, (2) ongoing staffing costs, (3) space charges, and (4) overheads (Table 2). All staffing hours were calculated from actual values observed in the original RCT and differentiated by academic level. Other values where modeled have been explicitly stated in Table 2, along with the assumptions made. Where appropriate, results have been expressed as the difference between F2F and BL due to commercial sensitivities.

**Table 2:** Description of cost categories.

Cost	F2F	BL	Description
Transition costs	No	Yes	Staff time for creating YouTube resources, tailoring content for BL approach, and designing activities.
Ongoing staff costs	Yes	Yes	Staff time for regular preparation for incoming students, teaching, unit-coordinating, and marking assessments.
Staff on-costs	Yes	Yes	Staffing salaries were assumed to be the highest step within the academic level as per the Monash University Enterprise Agreements [30]. Further on-costs include superannuation contributions, payroll taxes, loading, and levies. Academic staff (professors and associate professors) receive 17% superannuation contribution, with total on-costs of 39.62%. Casual staff receive 9.25% superannuation, hence total on-costs for casual staff is 15.43%.
Space costs	Yes	Yes	Space costs for classroom activities are dependent on seating capacity and AV capabilities, based on Monash University rate charges to faculties. Seating requirements were known from the RCT; however, AV capabilities were modeled as requiring full AV and digital projection. Number of classroom hours booked for each method was obtained from the university online timetable system records.
Overhead costs	Yes	Yes	The total operating overheads were modeled at 37% in line with Monash University’s Project Costing and Price Model. Central overheads were calculated at 22% (allocation of corporate services costs: finance, HR, IT, & corporate services). Faculty overheads were valued at 10% for the allocation of professional staff involved in the general support of students including student services, IT support for students, and research activities. Other overheads included 5% allocated to the general costs of running support activities.

### Sensitivity Analysis

We included a multivariate sensitivity analysis around permutations to the key variables. Guided by available literature, scenarios were constructed around increased transition costs and increased staffing requirements for running the BL format. Thus, the robustness of our economic model was tested, allowing the reader to tailor the findings to different educational settings.

## Results

### BL and F2F Approach Delivery Inputs

The staffing profile, wage, and difference in hours attributed to the delivery of each method are detailed in Table 3 below. Where there is a difference in hours, it is expressed as F2F hours minus BL hours; that is, positive values indicate fewer BL hours.

Space charges for the F2F and BL teaching methods were identical. Both methods had booked rooms seating 180 people (AUD $131.34/hr) for 28 hours and rooms seating 50 people (AUD $76.61/hr) for 210 hours. Major updating of the curriculum content and materials was considered required at 5 years and estimated at 160 hours of staff time for both F2F and BL approaches.

The cost of transitioning from the F2F to BL approach is the result of staffing time plus on-costs, valued at AUD $38,186. The difference in delivery costs is AUD $12,514 in favor of the BL method. The lower BL delivery cost is the result of lower preparation and direct teaching costs compared to the F2F method.

**Table 3:** Staffing profile with difference in hours for F2F and BL approaches.

Cost	Academic level	Rate, AUD	Difference (F2F hrs - BL hrs)
Transition costs (creating YouTube videos, tailoring content, and activities)	Professor	$84.23/hr	-40 hrs
	Associate Professor	$72.04/hr	-120 hrs
	Casual staff with PhD	$47.57/hr	-100 hrs
	Casual staff no PhD	$39.78/hr	-160 hrs
Total hour difference for transition costs			-420 hrs
Preparation costs (getting ready for each class and regular minor updating)	Professor	$84.23/hr	5 hrs
	Associate Professor	$72.04/hr	15 hrs
	Casual staff with PhD	$47.57/hr	12.5 hrs
	Casual staff no PhD	$39.78/hr	0 hrs
Direct teaching (classroom activities)	Professor	$84.23/hr	5 hrs
	Associate Professor	$72.04/hr	15 hrs
	Casual staff with PhD	$47.57/hr	12.5 hrs
	Casual staff no PhD	$39.78/hr	20 hrs
Unit coordination	Associate Professor	$72.04/hr	0 hrs
Marking and examining	Professor	$84.23/hr	0 hrs
	Associate Professor	$72.04/hr	0 hrs
	Casual staff with PhD	$47.57/hr	0 hrs
	Casual staff no PhD	$39.78/hr	0 hrs
Total hour difference for ongoing costs			85 hrs

### Cost Effectiveness

The BL method was less costly and more effective to operate than the F2F approach. The ICER result comparing F2F to BL was -$1.10, indicating that to operate the BL model, there is a saving of $1.10 per student per increase in QASE above the QASE of the F2F method. Independent ICER calculations of F2F and BL compared to baseline found that BL was 24% more cost-effective to operate than F2F. That is, the cost of achieving a statistically similar Berlin score for the same number of students is 24% less in the BL method, excluding transition cost. Given that the BL approach is slightly more effective than the F2F approach, the BL approach would have to cost 18% more than the F2F approach to run for there to be no difference in ICER between the two approaches.

### Break-Even Analysis

**Figure 4:**
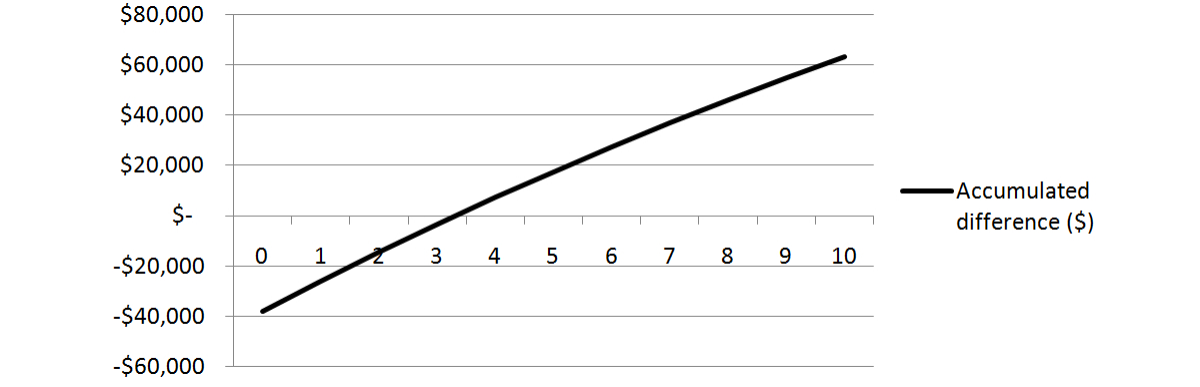
The accumulated difference in the PV of cost between F2F and BL across 10 years.

The PV difference in cost between F2F and BL approaches, including the transition costs to the BL model, are shown in Figure 4. The graphed line demonstrates the sunk cost of the transition to the blended learning approach, the cost of which is recovered by the lower running cost of the BL method in the third year. Subsequent years show a relative saving using the BL method, with PV savings of approximately AUD $17,000 after 5 years and AUD $63,000 after 10 years.

### Sensitivity Analysis

A multivariate sensitivity analysis was conducted with scenario variations to ongoing staffing costs and mean Berlin score (Table 4). Results are reported as the percentage difference in the independent F2F and BL ICER values. Two scenarios of educational effectiveness with the BL approach were simulated, that of mean Berlin scores as per the RCT and another with scores equivalent to the F2F method. The results show the BL format is more cost-effective to operate up to staffing levels 20% higher than F2F, using the RCT mean Berlin score.

**Table 4:** Multivariate sensitivity analysis adjusting staffing levels and mean BL Berlin score.

Scenario variation	Adjustment made	ICER % difference by mean BL Berlin score^a^	
		Effectiveness as per RCT	Equivalent effectiveness
Current model	Nil	24%	10%
Same preparation time	Increased BL staff time by 32.5 hrs	22%	7%
Same teaching time	Increase BL staff time by 52.5 hrs	18%	3%
Same preparation and teaching time	Increase BL staff time by 85 hrs	15%	0%
BL staff time 20% higher than F2F	Increase BL staff time to 20% higher than F2F for preparation and teaching	8%	-9%
BL staff time 50% higher than F2F	Increase BL staff time to 50% higher than F2F for preparation and teaching	-4%	-23%

^a^Expressed as BL in relation to F2F. Positive values indicate BL is more cost-effective; negative values indicate F2F is more cost-effective.

Table 5 explores various transition cost scenarios and the resulting transition cost. Using the scenarios from Table 4, the analysis shows changes in 5-year accumulated PV as a result of modifying ongoing and transition costs. Negative values indicate that, at 5 years, the savings made from running the lower cost BL format have not yet overcome the transition cost. In fact, in the scenarios in the last 3 columns of the table, the cost of running the BL program is greater than or equal to that of the F2F program, and as such will never recover their transition costs.

**Table 5:** Multivariate sensitivity analysis adjusting transition costs and ongoing cost scenarios.

Transition scenario	Transition cost, AUD $	5-year accumulated PV difference in AUD $ by scenario (F2F - BL - transition cost)					
		Current model	Same preparation time	Same teaching time	Same preparation and teaching time	BL staff time 20% higher than F2F	BL staff time 50% higher than F2F
Current model	38,186	17,523	1,673	-22,653	-38,186	-87,655	-161,382
Added 210 hrs of IT support	50,931	4,779	-11,072	-35,398	-50,931	-100,400	-174,127
Break-even at 5 years	55,710	0	-15,851	-40,177	-55,710	-105,179	-178,906
Break-even at 10 years	101,499	-45,789	-61,640	-85,966	-101,499	-150,968	-224,695

## Discussion

### Principal Findings

This research aimed to determine the cost-effectiveness of an F2F and BL approach for EBM training within a medical program. The training had known outcomes evaluated through a sufficiently powered randomized controlled trial. The ICER results indicate that the BL approach provided an improved cost-effectiveness proposition from the perspective of the educational institution—costing less to train student clinicians to an equivalent level of competency. Cost-effectiveness results should be interpreted together with the break-even point of 3 years, as it is after this point that savings are realized by the institution. In addition to this, the findings by Ilic et al [12] indicated that the blended learning approach had the added benefit of increasing student attitudes toward EBM and self-reported use of EBM in clinical practice.

Given that blended learning can take a wide variety of formats, the findings of this study show that low-cost resources such as YouTube and student self-directed activities are cost-effective at improving EBM. This may be in part due to the nature of EBM teaching, as opposed to other aspects of medical education that may tend to utilize more costly mediums such as animations or virtual patients, which few medical schools can afford to create [31]. In fact, many costly features such as animations, high-quality video products, and excessive multimedia have little added value and may actually impede learning [20]. Additionally, given that the online learning utilized either currently owned or free access resources, there were no new costs attributed to software or licensing. Prior to using the BL approach in EBM, lecture material and online resources were already available through the online learning management system. In the RCT, the combination of using simple BL formats and staff having prior experience with learning technology may have helped to exclude transition costs such as IT support, consultation, and piloting. Despite this, the impact of support roles in transitioning learning format appears to be highly situation dependent, given that a study on developing an e-module found that approximately one third of development time was attributed to administrative or technical staff [19]. The impact of IT support costs on our cost model was tested in the sensitivity analysis, calculated as an additional 210 hours of staff time to transition. Despite significantly increasing the cost to transition, over 5 years, this cost was recovered by the savings in operating costs.

The differences in operating cost between the two approaches is dependent on staffing time, specifically the preparation and teaching time. The lower values in the BL approach can be attributed to greater emphasis on peer-to-peer learning and self-directed activities. Staff members act as facilitators rather than tutors, guiding students to resources rather than direct teaching. In contrast, previous studies have highlighted the significant increase in staffing time associated with online education methods [19,32]. Increases appear to be the result of using email, discussion forums, and online chat sessions, 75% of which occur outside regular working hours [19]. Thus, it is likely that not all BL formats would be cost-effective, and educators should carefully design their learning models around staff time, as this is the most significant cost driver. This is reflected in the sensitivity analysis showing that large increases in staffing hours above the traditional approach result in unfavorable ICER values and an inability to recover the transition cost. As would be expected, the change in teaching method had no impact on unit coordination, marking, or examination costs.

The BL format used in this study did not reduce the face-to-face classroom hours. Rather, there was an emphasis on how existing resources were used, how time was spent outside of class, and changing what activities were completed in class. That is, the cost reduction to achieve the same or better outcome observed in this study, is naturally a reflection of productivity gains around resources used based on enhanced teaching formats. As a result, the cost-effectiveness found in this approach may not be generalizable to institutions seeking to reduce student contact hours or promote distance education. Additionally, this study benefited from economies of scale as the EBM program educates approximately 500 students across multiple campuses. Small increases in education effectiveness are magnified when the effect is across a large number of students, resulting in favorable ICER and break-even results. This multiplication effect also applies to staff time, with a YouTube video produced by one person replacing the work of many people. If preparation time is reduced by just 20 minutes for a session, across 5 sites and 10 sessions the reduction in preparation adds up to over 15 hours. Thus, when designing a cost-effective program, it appears pertinent to consider not only what aspect of learning is being changed, but how many people the change will influence.

There may be concerns over the acceptability of a BL approach from a student or industry perspective. Social demands are driving information and communication technologies to be more commonplace and publically acceptable. It is conceivable that although the BL approach was accepted by students within this study, that this may have been a function of time, and the same approach may not have been acceptable in the preceding years. The concept of pre-class homework is not new, and changing the format does not necessarily solve the inherent issue of student adherence [20]. It remains unknown how different formats of BL will impact participation and outcomes [20]. Regardless, from the perspective of the student, pre-class activities require additional time and may be more or less efficient than traditional approaches [20]. To consider the student perspective on costs, a cost-benefit analysis using “willingness to pay” may be used. Such an analysis would help to consider student perceived value and inform changes in fee structure associated with changes in learning format.

### Limitations

The generalizability of this study to other populations, as a result of the specific approach to BL used, have already been discussed. However, it is also necessary to discuss the limitations of the methodology used, which may influence the strength of the findings. The first limitation is the use of the ICER as a measure of cost-effectiveness. As mentioned, the standard use of the ICER does not include the investment cost of transitioning learning format. It is possible to include the investment cost into the ICER calculation for the first iteration only, as this is the period when the cash flow occurs, when considering the investment as a prospective cost. However, the ICER would not be representative of other periods. By excluding the transition cost, the ICER presented in this study reflects the ongoing operational cost-effectiveness from the second iteration onwards and must be interpreted together with the break-even analysis. Thus, programs with high set-up costs and low running costs, which may be typical of many blended learning formats, are shown to be more cost-effective by the ICER. However, the blended format in the RCT had relatively low set-up costs, which have been roughly estimated by the course developers to be similar to the cost of transitioning from a blended approach to a traditional approach. For the same reasons, the BL 5-year major curriculum update was estimated to be the same as F2F. It is logical to infer that formats with high set-up costs will incur high updating costs; however, within an educational institution, updating is often limited by time availability. Another consideration with the ICER is that it assumes a linear increase in EBM expertise. That is, the assumption that an increase in Berlin score from 30% to 40% represents the same increase in EBM expertise as an increase from 70% to 80%. There is insufficient literature on the Berlin Questionnaire to draw conclusions on the proficiency distribution, and while this does not preclude the use of the ICER calculation, it should be considered when interpreting the results.

In the RCT itself, the quality of the teacher within both methods may also influence the costs and the educational outcomes. Within the study, this potential variable was controlled for by using the same teaching staff across both modes of education. However, in using the same staff, there is the risk that staff may inadvertently bias the results in favor of the teaching model that they are more skilled or enthusiastic about [33]. The scope of research does not include variations in the savings made by the institution. If the blended and face-to-face approaches were offered with different fee structures, the total value proposition from the educational institution will change. Less tangible benefits from the modes of delivery, such as feelings of connectedness often associated with face-to-face learning [34], flexibility [20], and other measures of the full educational experience have not been calculated, and remain as unknown for both of the educational approaches utilized [35-37]. And finally, there is the assumption that increasing the number of EBM trained practitioners, competent in incorporating best evidence, with patient values and clinical practice, actually makes a difference to patient outcomes.

No approach to cost analysis in medical education is perfect; however, being transparent about the approach used is an important step forward considering the current state of the literature. Here, we feel we have been as transparent as possible about the costs of the components that made up the various approaches to medical education described. The most common errors in cost analyses include omitting hidden costs and paying insufficient attention to the main costs. In this regard, we feel we have gone to considerable lengths to seek out and account for hidden costs and that we have paid most attention to the main costs—that is, the costs of faculty and resources.

### Conclusions

Under the study settings, a blended learning approach to training practitioners to be competent in applying evidenced-based medicine was more cost-effective to operate than the traditional face-to-face model. Furthermore, the BL approach resulted in significantly greater increases in student attitudes toward EBM and self-reported use of EBM in clinical practice. When taking into account the cost of transitioning to the new format, the benefit of the cost-effectiveness is realized by the institution only after the third operational year. The primary drivers of cost-effectiveness were the low-cost online resources chosen, decreased staffing levels, and economies of scale. Implementing BL is not without its risks though and requires a significant investment cost in tailoring the teaching and learning resources to the Web-based environment during the transition to this approach. Using a BL approach will not necessarily be cost-effective, and consideration should be given to the blend utilized, staff expertise, and the educational setting. Health professions’ education and educational research has developed into a respectable scientific discipline due to the shift toward scientific rigor and peer-review [38,39]. To maintain its relevance and accountability and to improve the adoption of new educational approaches and innovations, the next cultural shift in this field needs to be toward fiscal responsibility alongside learning outcomes, such as measuring outcomes of cost-effectiveness alongside measures of educational outcomes and the learning experience. The purpose of this shift is not to cut costs or to increase spending but simply to improve value.

## Abbreviations

BL: blended learning
EMB: evidence-based medicine
F2F: face-to-face
ICER: incremental cost-effectiveness ratio
MD: mean difference
PV: present value
QASE: quality-adjusted students educated
RCT: randomized controlled trial
